# Geographical Variation in Body Size and the Bergmann’s Rule in Andrew’s Toad (*Bufo andrewsi*)

**DOI:** 10.3390/biology11121766

**Published:** 2022-12-06

**Authors:** Ying Jiang, Li Zhao, Xiaofeng Luan, Wenbo Liao

**Affiliations:** 1 School of Ecology and Nature Conservation, Beijing Forestry University, Beijing 100083, China; 2 Key Laboratory of Southwest China Wildlife Resources Conservation (Ministry of Education), China West Normal University, Nanchong 637009, China; 3 Key Laboratory of Artificial Propagation and Utilization in Anurans of Nanchong City, China West Normal University, Nanchong 637009, China

**Keywords:** environmental variation, body size, age, Bergmann’s rule, skeletochronology

## Abstract

**Simple Summary:**

Understanding variations in the morphology and age of animals along a geographical gradient may aid in our comprehension of the evolution of these animals. In this view, we studied variation in the age and body size of Andrew’s toad (*Bufo andrewsi*) across 31 populations along a geographical gradient. The results revealed that along with a decrease in the annual mean temperature, the age structure increased, whereas body size did not indicate an increasing trend, showing no support for Bergmann’s rule. Precipitation seasonality negatively correlated with longevity and mean age, whereas precipitation of the driest month positively correlated with body size. Moreover, we also found that UV-B seasonality positively correlated with age structure traits and body size. The present study provided critical cues that explain the considerable variability observed in the ecogeographic patterns among Andrew’s toads.

**Abstract:**

Environmental variation likely modifies the life-history traits of vertebrates. As ectothermic vertebrates, it is possible that the body size of amphibians is impacted by environmental conditions. Here, we firstly quantified age and body size variation in the Andrew’s toad (*Bufo andrewsi*) across the Hengduan Mountains. Then, we examined the environmental correlates of this variation based on the literature and our unpublished data on the age and body size of the Andrew’s toad from 31 populations distributed in southwestern China. Although our analysis revealed significant variations in age and body size across *B. andrewsi* populations, neither latitude nor altitude correlated with this variability in age and body size. We found that age at sexual maturity, mean age, and longevity increased with decreasing annual mean temperature, whereas age at sexual maturity increased with decreasing temperature seasonality, implying that temperature was a crucial habitat characteristic that modulated age structure traits. Moreover, we revealed positive associations between age structure and UV-B seasonality, and negative relationships between both mean age and longevity and precipitation seasonality. We also found that body size increased with increasing precipitation in the driest month and UV-B seasonality. However, body size did not covary with temperature, signifying no support for Bergmann’s rule. These findings help us to understand amphibians’ abilities to adapt to environmental variation, which is particularly important in order to provide a theorical basis for their conservation.

## 1. Introduction

Environmental variations can impose pressures on an animal’s physiology [1,2,3], phenology [4,5,6], morphology [7,8,9,10,11,12,13,14,15,16,17,18,19], distribution [20], and life-history strategies [21,22,23,24,25,26]. The ecogeographic patterns of covariation between biological traits and environmental variables [27] provide opportunities to assess the adaptions of animals in response to the selection pressures imposed by significant variations in temperature, precipitation, and associated microclimate variations [28,29]. The variation in life-history traits, such as age structure and body size, across environmental gradients is one of the most frequently studied ecogeographic patterns [30,31,32].

Body size is a key life-history trait [33,34,35,36,37] that is affected by some factors, including resource consumption, interactions, population dynamics and community assembly in different environments [38,39,40,41]. Thus, body size should be associated with metabolic rate, population density, longevity, and geographic range (see [38,42,43]). Identifying the environmental factors that affect body size variation among populations is important in order to understand how animals adapt to abiotic environments by changing their phenotypic plasticity [22,44,45,46,47,48].

A well-known ecogeographic pattern of variation in body size is Bergmann’s rule, which describes the tendency for endotherms to be larger in colder conditions at high latitudes or altitudes [49]. The potential mechanism underpinning Bergmann’s rule may be a reduction in heat loss due to the low surface-to-volume ratio of larger individuals (the heat conservation hypothesis) [49,50,51]. When applying Bergmann’s rule to ectothermic vertebrates which neither produce nor conserve heat [52], the conclusions remain ambiguous at either interspecific or intraspecific levels [53], for example, some taxa follow this rule [45,54], other taxa do not follow this rule [22,55] and many taxa show the inverse of Bergmann’s rule [56,57,58]. In other words, there is no consensus on the generalization of the Bergmann’s clines for ectothermic vertebrates and further research is required.

In view of this ambiguity, studies on body size variation among ectotherms have proposed three main hypotheses: the water supply hypothesis [59], the hibernation hypothesis [60], and the heat balance hypothesis [61]. These hypotheses emphasize the effects of environmental factors (e.g., water deficits and temperature seasonality) on growth rates and sexual maturity, which subsequently affect body size. For instance, individuals living in harsh environments devote more time and energy to growing, which causes them to become older and to grow larger, and, thus, these individuals are of a larger body size [62]. Furthermore, some species display sex-specific relationships between environmental factors and body size because males and females suffer distinct selection pressures in the same environment [63,64,65,66,67]. As such, the extent to which these hypotheses explain the ecogeographic patterns of body size variation remains uncertain.

The anuran is an ideal model for exploring the influence of environmental changes on body size, since their highly permeable skin, unshelled eggs, low vagility, and frequent territoriality are particularly vulnerable to environmental stresses (e.g., temperature, precipitation, and ultraviolet radiation) [68]. However, few studies exploring body size variation have been conducted, of which only nearly 5% of all amphibian species have been studied, and there is much controversy as to whether the effects of environmental changes on body size variation support Bergmann’s rule [45,55,57,61,65,66,67,68,69,70,71,72,73,74,75,76,77,78,79,80,81,82]. Although the effect of temperature on body size is contested, precipitation, as a proxy for productivity, has been found to affect the body size of frogs [24,83]. One possible explanation is that precipitation affects the abundance of food resources, humidity, and the length of the breeding season for anurans [84]. Therefore, individuals living in environments with less precipitation should display a shorter breeding season and obtain fewer resources, and will subsequently possess a smaller body size. Temperature and precipitation are not the only factors that affect the growth and development of many amphibian species, but other environmental factors, such as ultraviolet-B (UV-B) radiation, can also have different effects on the growth and development of amphibians [85]. For example, some studies have found that high UV-B radiation can damage DNA, resulting in increasing d [86,87], whereas inadequate levels of UV-B radiation may result in decreased head width, vertebrae length, and femur length [88]. Whether the final body size of the amphibian is the consequence of a unique main factor or several factors acting synergistically remains contested. In this case, a detailed study on body size variation across populations is necessary.

To examine the main driving force of body size variation, a species that is distributed across a wide geographic range may be the most ideal model, as different populations exist along geographical gradients and are consequently exposed to different climatic and environmental conditions. The Andrew’s toad (*Bufo andrewsi*) is not endemic to China and is widely distributed in the Hengduan Mountains, China, with altitudes ranging from 750 m to 3500 m [24,89]. Previous studies have investigated the life-history traits, male mating choice, testes mass, organ size (e.g., heart, lung, gallbladder, livers, spleen, kidneys, and digestive tract), and population genetic structure of this species [24,45,90,91,92,93,94,95,96]. However, studies on body size variation have investigated only a few populations [22,45]. Here, we use data on mean body size and age from our previously published paper [24] and our unpublished data to explore how environmental changes (i.e., temperature, precipitation, and UV-B radiation variables) affect the body size variations of *B. andrewsi*. The present study aimed to (1) examine the differences in age structure and body size among populations, (2) test the applicability of the Bergmann’s rule in *B. andrewsi*, and (3) characterise the associations between environmental factors, age structure, and body size. This study would help to elucidate the adaption of amphibians to environmental changes through variations in age structure and body size responding to changes of environmental stress.

## 2. Materials and Methods

### 2.1. Data on Body Size and Age Estimation

**Figure 1:**
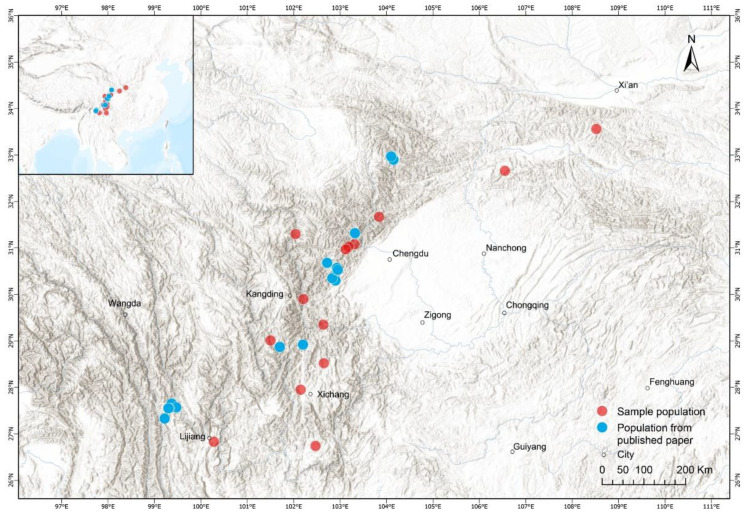
Geographic distribution of the studied populations for *B. andrewsi* at Hengduan Mountains in western China.

To explore the body size variation of *B. andrewsi* along its environmental gradients, we captured 309 males and 103 females from 14 populations between 2017 and 2019. The populations encompassed a wide range of the geographic distribution of this species. We diagnosed the toads as *B. andrewsi* based on their morphological key characteristics (body length and body colour) and distribution ranges [89]. Each population was not equidistant from the others (Figure 1). For all populations, individuals were captured by hand on spawning sites at night. After confirming whether the individuals were adults by directly observing secondary sexual traits, we used callipers to measure the snout-vent length (SVL) as an index of body size to the nearest 0.01 mm. Prior to being released at the collection site, the second phalange of the longest finger of the right hindlimb of all individuals were removed and preserved in 4% neutral buffered formalin for subsequent age estimation.

The skeletochronology was used to estimate the ages of each sampled individual [22]. To produce histological sections for age determination, we used paraffin sectioning and Harris’s haematoxylin staining (see details in [66,97]). With a LEITZ dialux 40 microscope, we selected the cross-sections of the phalanx that had the smallest medullar cavity and the thickest cortical bone (13 m thick) to count the lines of arrested growth (LAG). To take photos of the best portions, we utilized a Motic BA300 digital camera mounted on a Moticam2006 light microscope with a 400× magnification. When determining age in all samplings, we considered the effect of endosteal resorption, false, and multiple lines on the accuracy of age determination.

We then extracted reliable data on body size, age, and the sample coordinates of 17 populations from the published literature [24], in which the same standard measurements were performed. Considering that all toads captured at the spawning sites provided for the age distribution of the reproductive population, it was reasonable to use the minimum age of adult toads as an estimate of age at sexual maturity in a population and the maximum age as an estimate of longevity. A total of 2240 toads (1663 males, 577 females) were estimated for their ages, with measurements of body size (Table S1).

### 2.2. Environmental Predictors

To explore the effects of environmental changes on the body size variation of *B. andrewsi*, the bioclimatic and UV-B variables were used as environmental predictors, which were found to be influential for the anurans’ survival [86,98,99,100]. We obtained bioclimatic data from WorldClim v2 [100] and UV-B data from the glUV dataset v1 [99] and extracted those variables for the sampling sites using ArcGIS 10.8 [101]. Then, to avoid high collinearity among bioclimatic variables and UV-B variables, respectively, we used Pearson’s correlation tests to analyse their correlations and exclude high-related variables (Figure S1) [85,102]. Five bioclimatic variables and two UV-B variables were retained for subsequent analysis, including annual mean temperature (a measure of heat in the environment), temperature seasonality (an indicator of energy predictability), annual precipitation (a measure of water availability), precipitation seasonality (an indicator of water predictability), precipitation of the driest month, UV-B seasonality, and mean UV-B of the lowest month (Table S2).

### 2.3. Statistical Analysis

All statistical analyses were conducted in R 4.2.0 [103]. Prior to analyses, continuous variables were log_10_-transformed to meet the normality assumption.

To explore geographical variation in age structure (i.e., age at sexual maturity, longevity, and mean age) among the 14 populations, we first used the R package ‘lme4′ [94,104] to implement the generalized linear mixed models (GLMMs) with age as the dependent variable, sex as a fixed factor, and the population as a random factor. We then conducted GLMMs with age as the dependent variable, altitude and latitude as fixed factors, and population as a random factor to examine the effect of geographical gradients on age. Furthermore, we performed those models again with sex added into the models as a covariate to control for the effect of sex on age.

To investigate differences in body size between males and females among populations, we treated body size as the dependent variable, sex as a fixed factor, and population as a random factor. We further tested for variation in body size among populations when controlling for the effect of age on body size, age was added into the model as a covariate together with sex × age (fixed effect) and age × population (random effect). Sex differences in growth rates would be suggested by a significant sex–age interaction. To estimate the effect of geographical gradients on body size, a GLMM was used. Here, population was used as a random factor, latitude and altitude as fixed factors, and sex as a covariate.

To test the hypothesis that age covaries with environmental variables, we implemented several general linear models (GLMs) with age as the dependent variable, bioclimatic and UV-B data as independent variables, and sex as a covariate. We conducted the test for the effects of environmental factors on body size using a GLM in which body size was considered as a dependent variable, bioclimatic and UV-B data as independent variables, and sex and mean age as covariates.

## 3. Results

### 3.1. Geographical Variation in Age

GLMMs showed that age at sexual maturity differed significantly between the sexes (F = 15.560, *p* = 0.002), with females ageing later at sexual maturity than males, but not among the 14 populations (AIC = −7.961, *p* = 0.162). Meanwhile, longevity differed significantly among the 14 populations (AIC = −28.933, *p* = 0.009) but not between the sexes (F = 0.595, *p* = 0.454). Moreover, age at sexual maturity and longevity did not increase with increasing altitude or latitude (Table 1). Controlling the effect of sex, age at sexual maturity and longevity still displayed no correlation with altitude (age at sexual maturity: F = 0.182, *p* = 0.678; longevity: F = 0.003, *p* = 0.952). Although mean age differed significantly among 14 populations (GLMM: AIC = −29.019, *p* = 0.017) and between males and females (F = 6.652, *p* = 0.023; Table 2), the effects of altitude and latitude on mean age remained insignificant after correcting for sex effect (Table 1).

### 3.2. Geographical Variation in Body Size

GLMM indicated that mean body size differed significantly among the 14 populations (AIC = −80.809, *p* = 0.011) and between the sexes (F = 79.056, *p* < 0.001). Females always had larger body sizes than males (Table S1). When controlling the age effect (F = 0.164, *p* = 0.690), differences in body size remained significant among 14 populations (AIC = −71.758, *p* = 0.015) but not between the sexes (F = 1.455, *p* = 0.248). The non-significant interaction effects of sex and age on body size revealed that the relationship between body size and age (≈growth rate) did not differ between the sexes (F = 0.275, *p* = 0.608). The age × population interaction was also non-significant (AIC = −75.702, *p* > 0.5), indicating that the growth rate did not differ among the populations. Contrary to our prediction, the effects of altitude and latitude on mean body size were not significant across 14 populations, regardless of whether the effects of sex were controlled (Table 1).

### 3.3. Effect of Environmental Factors

For 31 populations, age at sexual maturity, longevity, and mean age were negatively correlated with annual mean temperature (age at sexual maturity: t = −2.644, *p* = 0.011; longevity: t = −2.548, *p* = 0.014; mean age: t = −2.983, *p* = 0.004), indicating that individuals of populations living in lower-temperature environments matured sexually later, had older mean age and lived longer than those living in higher temperature environments (Table 2). Meanwhile, although age at sexual maturity did not change with variation of precipitation, mean age, and longevity were negatively associated with precipitation seasonality (Table 2). Significant and positive trends were detected between age at sexual maturity (t = 2.769, *p* = 0.008), longevity (t = 3.081, *p* = 0.003), mean age (t = 2.902, *p* = 0.005), and UV-B seasonality. Moreover, mean UV-B of the lowest month showed a negative effect on age at sexual maturity (t = −2.232, *p* = 0.029).

For 31 populations, we found that mean body size cannot be predicted by temperature when the effects of sex and age were controlled, which was inconsistent with the prediction of Bergmann’s rule that larger individuals lived at lower temperature (Table 2). However, mean body size was positively associated with precipitation of the driest month (t = 2.339, *p* = 0.023) and UV-B seasonality (t = 2.506, *p* = 0.015).

## 4. Discussion

Our results provided significant evidence for body size variation in *B. andrewsi* across populations in response to environmental conditions. Inconsistent with the predictions, we did not find any significant effects of altitude and latitude on variation in age characteristics and body size among 14 populations. Age at sexual maturity, longevity, and mean age increased with decreasing annual mean temperature in 31 populations. Populations living under low-temperature conditions have earlier sexual maturity, better longevity, and larger mean age than populations living under high-temperature conditions. However, the non-significant relationship between body size variation and temperatures fails to support Bergmann’s rule when correcting age and sex effects. Moreover, a negative relationship between age at sexual maturity and temperature seasonality indicated that individuals living in fluctuating temperature environments mature earlier. Animals reproduce earlier (younger, and with smaller size) when predators are present in their environment [33]. Similarly, our results demonstrated significant negative correlations between both longevity and mean age and precipitation seasonality but showed marked positive correlations between age characteristics and UV-B seasonality. Body size increased with increasing precipitation of the driest month and UV-B seasonality. In what follows, we discussed our findings in association with what was previously known from intraspecific anuran studies.

Like those of most other ectotherms [67,105,106], anuran life-history traits (e.g., age at sexual maturity, mean age, and longevity) vary with environmental conditions [66,82,107,108]. For instance, populations experiencing longer growth seasons have younger ages at sexual maturity, mean age, and longevity than populations experiencing shorter growth seasons in previous studies that discussed geographical variation in *B. andrewsi* age structure [22,45]. In this study, we found that low temperature led to older age at sexual maturity and mean age and longer longevity across 31 populations. The observed increase in age at sexual maturity, mean age, and longevity with decreasing annual mean temperature may reflect food resources and predation risk. Indeed, low temperatures led to food limitation because invertebrates are regarded as the major food resources of anurans that have decreased in quantity [109]. Moreover, predation risk becomes weak at low-temperature environments compared with high-temperature environments [110]. These risks are expected to increase juvenile mortality, and juveniles are likely to need a longer time to reach adulthood, leading to later age at sexual maturity and higher mean age [22,106,111]. Meanwhile, the toads living under lower temperature conditions devote more energy to somatic growth and survive longer than those living under higher temperature conditions because of the increased predation rates at high-temperature environments. Hence, variations in environmental factors determine the direction of age structure variations across geographical gradients.

Interestingly, a previous study on the growth rate in *B. andrewsi* suggested that females have a larger growth rate than males when they live shorter growth seasons whereas males have larger growth rates than females when they experience longer growth seasons [22]. For both sexes, high-altitude populations have a smaller growth rate than lower-altitude populations [45]. In this study, we found that the effects of the interaction of age and sex on body size were non-significant among populations, suggesting that males and females had similar growth patterns. Moreover, the non-significant effects of the interaction of age and population on body size suggested that high-altitude populations did not have a smaller growth rate, and this result differed from the previous findings [45].

Previous studies have shown that body size variation and Bergmann’s rule along geographic gradients were determined by three main parameters, namely, age at sexual maturity, longevity, and growth rate [22,45]. In the case of Bergmann’s rule, later age at sexual maturity and better longevity can play greater roles in promoting an increase in body size than slower growth in promoting a decrease in body size. The converse Bergmann’s cline is observed among populations within a species when growth rate is contained so that any prolonged time spent on growth fails to compensate for the effect of slow growth on body size [45]. In this study, body size did not increase with altitude and decreasing temperature among all populations. The age at maturity and longevity were also not correlated with altitude but increased with decreasing annual mean temperature. These findings suggested that body size variation that does not follow Bergmann’s rule, which was attributed to the fact that the increase in body size resulting from later age at sexual maturity and better longevity did not have greater roles than the decrease in body size resulting from slower growth rate. This result is inconsistent with other studies on body size variation and environmental conditions in ectotherms [26,45,55].

Life-history traits are often related to precipitation in anurans, as many studies have found an increasing trend for both body size and age with decreasing precipitation [112,113]. The observed negative relationship between longevity and mean age and precipitation seasonality of *B. andrewsi* was inconsistent with the prediction that better longevity may occur in harsher environments [113]. We attributed this phenomenon to the fact that environments with high fluctuations in rainfall were not conducive to survival and result in shorter longevity and smaller mean age, because the toads cannot survive and reproduce without water. Furthermore, the positive relationship between body size and precipitation of the driest month was observed, which may indicate that the toads had more time and energy to devote to growth when in a resource-rich environment resulting from abundant water.

The cost of producing protective pigments, repairing cellular damage, or behaviourally avoiding UV-B radiation may delay individual growth under conditions with low levels of UV-B [114], whereas individuals with relatively high UV-B exposure show preferential allometric skeletal development of components due to calcitriol secretion [88]. Moreover, UV-B radiation may affect embryo survival and development in amphibians [87,88]. Our findings indicated that high values of UV-B seasonality had positive effects on the age and body size of *B. andrewsi*, suggesting that the toads would achieve better longevity and grow larger in regions with high seasonality in UV-B radiation. This condition may compel individuals to trade-off energy input and time investment into growth.

Geographical variation in body size in the toads is driven by genetic differences [115,116]. Although the responses of the size-related elements to environmental changes are common [28,117], potential genetic effects on body size variation in the toads across populations based on common garden experiments need to be considered.

## 5. Conclusions

In conclusion, we used data from 2240 individuals of the amphibian *B. andrewsi* across distribution ranges of the species in China to examine the effects of environmental changes on the body size and age structure of the species. The results of our study confirmed significant geographical variation in body size among the 31 populations. We failed to identify any correlations between both latitude and altitude and age or body size among *B. andrewsi* populations, which was contrary to our predictions. Nevertheless, we found that individuals from populations with lower temperatures matured earlier and had larger mean ages and better longevity than individuals from populations with higher temperatures. Moreover, a negative relationship between age at sexual maturity and temperature seasonality suggested that those who live in climates with temperature fluctuations tend to mature earlier. We also found that after removing the effects of sex and age, although body size increased in response to increasing rainfall in the driest month and UV-B seasonality, the non-significant link between body size and temperature did not follow Bergmann’s rule.

## Figures and Tables

**Table 1:** The influences of geographic gradients and population on variation in age and body size across 14 populations of Andrew’s toads (*B. andrewsi*) using GLMMs.

Source	Random			Fixed			
	VAR	SD	*p*	Estimate	df	F	*p*
Age at sexual maturity							
Population	0.010	0.099	0.203				
Residual	0.018	0.134					
Altitude				0.192	11.025	0.182	0.678
Latitude				1.752	11.149	2.009	0.184
Sex				0.196	12.994	14.965	0.002
Longevity							
Population	0.005	0.073	0.044				
Residual	0.005	0.069					
Altitude				−0.018	11.003	0.004	0.952
Latitude				1.643	11.196	4.297	0.062
Sex				0.018	12.954	0.476	0.503
Mean age							
Population	0.007	0.082	0.029				
Residual	0.005	0.072					
Altitude				0.134	11.046	0.178	0.682
Latitude				1.320	11.260	2.284	0.158
Sex				0.068	12.991	6.227	0.027
Body size							
Population	0.001	0.033					
Residual	0.001	0.025					
Sex				0.085	13.000	79.056	<0.001
Body size							
Mean age: Population	<0.001	0.018	1.000				
Population	0.001	0.034	0.015				
Residual	<0.001	0.018					
Sex				0.059	13.598	1.455	0.248
Mean age				−0.093	18.793	0.164	0.690
Mean age: Sex				0.061	14.565	0.275	0.608
Body size							
Population	0.001	0.035	0.009				
Residual	0.001	0.025					
Altitude				0.091	11.000	0.499	0.495
Latitude				0.074	11.286	0.044	0.838
Sex				0.085	12.928	78.151	<0.001

**Table 2:** The influences of environmental factors on variation in age and body size across 31 populations of Andrew’s toads (*B. andrewsi*) using GLMs.

	Variable	*β*	SE	*t*	*p*
Age at sexual maturity	Annual mean temperature	−0.236	0.089	−2.644	0.011
	Temperature seasonality	0.707	0.354	1.998	0.050
	Sex	0.225	0.041	5.458	<0.001
Age at sexual maturity	Annual precipitation	−0.909	0.651	−1.397	0.168
	Precipitation of the driest month	−0.078	0.175	−0.445	0.658
	Precipitation seasonality	−0.327	0.555	−0.589	0.558
	Sex	0.225	0.042	5.387	<0.001
Age at sexual maturity	UV-B seasonality	0.823	0.297	2.769	0.008
	Mean UV-B of the lowest month	−0.643	0.288	−2.232	0.029
	Sex	0.225	0.043	5.285	<0.001
Longevity	Annual mean temperature	−0.240	0.094	−2.548	0.014
	Temperature seasonality	0.575	0.373	1.542	0.128
	Sex	0.043	0.043	0.991	0.326
Longevity	Annual precipitation	−0.592	0.559	−1.060	0.294
	Precipitation of the driest month	−0.018	0.150	−0.118	0.906
	Precipitation seasonality	−1.618	0.476	−3.396	0.001
	Sex	0.043	0.036	1.202	0.234
Longevity	UV-B seasonality	0.928	0.301	3.081	0.003
	Mean UV-B of the lowest month	−0.558	0.292	−1.910	0.061
	Sex	0.043	0.043	0.998	0.322
Mean age	Annual mean temperature	−0.247	0.083	−2.983	0.004
	Temperature seasonality	0.429	0.329	1.306	0.197
	Sex	0.100	0.038	2.599	0.012
Mean age	Annual precipitation	−0.136	0.517	−0.263	0.794
	Precipitation of the driest month	−0.140	0.139	−1.012	0.316
	Precipitation seasonality	−1.658	0.440	−3.766	<0.001
	Sex	0.100	0.033	3.006	0.004
Mean age	UV-B seasonality	0.786	0.271	2.902	0.005
	Mean UV-B of the lowest month	−0.452	0.263	−1.721	0.091
	Sex	0.100	0.039	2.567	0.013
Body size	Annual mean temperature	0.015	0.025	0.607	0.546
	Temperature seasonality	0.029	0.094	0.306	0.761
	Sex	0.085	0.011	7.477	<0.001
	Mean age	0.053	0.037	1.450	0.152
Body size	Annual precipitation	−0.259	0.146	−1.778	0.081
	Precipitation of the driest month	0.092	0.039	2.339	0.023
	Precipitation seasonality	−0.094	0.139	−0.680	0.500
	Sex	0.092	0.010	9.149	<0.001
	Mean age	−0.019	0.037	−0.499	0.620
Body size	UV-B seasonality	0.191	0.076	2.506	0.015
	Mean UV-B of the lowest month	−0.126	0.071	−1.775	0.081
	Sex	0.089	0.011	8.238	<0.001
	Mean age	0.014	0.035	0.404	0.688

## Data Availability

The data presented in this study are available on request from the corresponding author. The data are not publicly available due to privacy or ethical restrictions.

## Supplementary Materials

The following supporting information can be downloaded at: https://www.mdpi.com/article/10.3390/biology11121766/s1, Table S1: Descriptive information about the study sites of Andrew’s toad (*B. andrewsi*), together with mean (±SD) body size and age characteristics of males and females; Table S2: Environmental variables compiled to depict environment gradients for Andrew’s toad (*B. andrewsi*); Figure S1: Pearson’s correlation tests for bioclimatic variables and UV-B variables.
