# Supplementary material for: Geographical Variation in Body Size and the Bergmann’s Rule in Andrew’s Toad (Bufo andrewsi)
Source: Biology (Basel). 2022 Dec 6;11(12):1766. doi: 10.3390/biology11121766 (PMC9775554; doi:10.3390/biology11121766)

Electronic Supplementary Material

Geographical Variation in Body Size and the Bergman's Rule in Andrew's Toad (*Bufo andrewsi*)

Ying Jiang [1,2,3], Li Zhao [2,3], Xiaofeng Luan [1,*] and Wenbo Liao [2,3,*]

[1]School of Ecology and Nature Conservation, Beijing Forestry University, Beijing 100083, China

[2]Key Laboratory of Southwest China Wildlife Resources Conservation (Ministry of Education), China West Normal University, Nanchong 637009, China

[3]Key Laboratory of Artificial Propagation and Utilization in Anurans of Nanchong City, China West Normal University, Nanchong 637009, China

*Correspondence: luanxiaofeng@bjfu.edu.cn (X.L.); liaobo_0_0@126.com (W.L.)

Table S1. Descriptive information about the study sites of Andrew's toad (*B. andrewsi*), together with mean (±SD) body size and age characteristics of males and females.

Study sites	Longitude (E)	Latitude (N)	Altitude (m)	Females SVL (mm)	Males SVL (mm)	Females mean age (years)	Males mean age (years)	Females age at sexual maturity (years)	Males age at sexual maturity (years)	Females longevity (years)	Males longevity (years)	Source
Gengda	103.31	31.08	1524	87.5±10.8 *n* = 10	67.3±5.6 *n* = 30	2.9±0.9 *n* = 10	1.9±0.6 *n* = 30	2	1	4	3	unpublished data
Anning	102.15	27.95	1522	73.1±8.1 *n* = 5	66.8±8.1 *n* = 23	2.4±0.5 *n* = 5	2±0.6 *n* = 23	2	1	3	3	unpublished data
Gucheng	100.28	26.83	2367	101.7±11.0 *n* = 4	79.6±8.6 *n* = 26	3.0±1.0 *n* = 4	2.7±0.9 *n* = 26	2	1	4	4	unpublished data
Hanyuan	102.64	29.35	864	75.6±11.4 *n* = 8	64.2±11.1 *n* = 26	2.8±1.1 *n* = 8	2.3±1.1 *n* = 26	2	1	4	3	unpublished data
Jinchuan	102.04	31.3	2078	77.0±4.9 *n* = 5	72.9±4.4 *n* = 26	2.2±0.7 *n* = 5	2.3±0.6 *n* = 26	2	2	3	4	unpublished data
Jiulong	101.5	29.01	2902	82.5±8.2 *n* = 11	67.8±6.0 *n* = 16	3.1±0.7 *n* = 11	2.7±0.7 *n* = 16	2	2	4	4	unpublished data
Luding	102.21	29.9	1477	73.2±7.9 *n* = 4	68±8.0 *n* = 21	3.3±1.0 *n* = 4	2.2±1.0 *n* = 21	3	1	4	5	unpublished data
Lamasi	103.18	31.03	1961	89.3±7.1 *n* = 13	70.6±4.3 *n* = 25	3±1.1 *n* = 13	2.3±0.7 *n* = 25	1	1	5	4	unpublished data
Maoxian	103.84	31.67	1553	85.4±7.2 *n* = 11	75.9±7.3 *n* = 19	2.3±0.7 *n* = 11	2.6±0.8 *n* = 19	2	2	4	4	unpublished data

Puxiong	102.65	28.52	1864	93.0±10.5<br>*n* = 5	70.6±8.9<br>*n* = 8	1.8±0.7<br>*n* = 5	2.4±0.7<br>*n* = 8	1	1	3	3	unpublished data
Shiziba	106.55	32.66	1651	104.8±14.9<br>*n* = 10	76.9±14.3<br>*n* = 26	3.7±1.3<br>*n* = 10	2.6±1.3<br>*n* = 26	3	1	5	5	unpublished data
Taiping	102.47	26.74	1916	92.1±11.5<br>*n* = 9	70.1±10.0<br>*n* = 22	2.3±0.6<br>*n* = 9	2.6±0.6<br>*n* = 22	2	2	3	4	unpublished data
Xunyangba	108.52	33.56	1393	79.0±7.5<br>*n* = 4	63.7±6.6<br>*n* = 28	6.3±1.7<br>*n* = 4	3.9±1.6<br>*n* = 28	4	2	9	6	unpublished data
Yinchangou	103.12	30.97	2153	92.6±10.7<br>*n* = 4	74.4±4.4<br>*n* = 13	4.3±0.5<br>*n* = 4	3.2±0.6<br>*n* = 13	4	2	5	4	unpublished data
Lingguan	102.9	30.3	760	94.2±5.2<br>*n* = 32	72.9±3.8<br>*n* = 47	3.3±0.9<br>*n* = 32	2.0±0.8<br>*n* = 47	2	1	5	4	Liao et al. 2015
Muping	102.83	30.35	1000	97.3±4.3<br>*n* = 26	74.1±4.3<br>*n* = 45	3.6±1.0<br>*n* = 26	2.1±0.7<br>*n* = 44	2	1	6	4	Liao et al. 2015
Yanjing	102.92	30.53	1390	98.8±4.9<br>*n* = 8	78.2±3.8<br>*n* = 18	4.1±0.9<br>*n* = 7	2.7±0.7<br>*n* = 15	3	2	5	4	Liao et al. 2015
Dengcigou	102.93	30.55	1690	99.7±4.9<br>*n* = 192	79.9±4.5<br>*n* = 361	4.2±1.1<br>*n* = 120	2.2±0.9<br>*n* = 229	3	2	7	6	Liao et al. 2015
Zhalangou	102.93	30.57	1800	101.0±3.8<br>*n* = 9	80.2±4.2<br>*n* = 29	4.3±1.4<br>*n* = 9	3.3±1.0<br>*n* = 11	3	2	7	5	Liao et al. 2015
Church	102.95	30.53	2100	103.2±4.4<br>*n* = 22	82.6±3.4<br>*n* = 30	5.2±1.2<br>*n* = 22	4.1±0.9<br>*n* = 22	3	3	8	6	Liao et al. 2015
Yaoji	102.72	30.68	2387	87.5±5.9<br>*n* = 7	74.3±6.1<br>*n* = 17	3.7±0.8<br>*n* = 7	4.1±0.1<br>*n* = 15	3	3	10	9	Liao et al. 2015
Muli	101.70	28.87	1710	84.9±1.3<br>*n* = 5	69.0±6.0<br>*n* = 12	4.2±1.1<br>*n* = 5	3.4±1.1<br>*n* = 12	3	2	6	5	Liao et al. 2015

Xingguqin	99.37	27.65	2768	97.4±5.6 *n* = 15	76.0±7.5 *n* = 203	7±2.3 *n* = 14	4.7±2.3 *n* = 197	3	1	12	11	Liao et al. 2015
Qibie	99.47	27.57	2028	96.8±7.4 *n* = 27	73.7±7.9 *n* = 167	5.6±1.9 *n* = 27	3.7±1.3 *n* = 167	3	1	10	9	Liao et al. 2015
Kegong	99.32	27.55	2422	95.9±7.8 *n* = 17	74.8±5.4 *n* = 67	6.2±1.2 *n* = 17	4.1±1.3 *n* = 65	3	2	7	7	Liao et al. 2015
Kegong	99.30	27.55	2328	89.8±4.4 *n* = 4	74.6±6.4 *n* = 110	7.6±1.7 *n* = 4	4.4±1.7 *n* = 110	3	2	10	9	Liao et al. 2015
Pantiange	99.22	27.33	2520	91.9±9.7 *n* = 13	72.6±4.8 *n* = 75	4.5±1 *n* = 13	3.6±1.3 *n* = 75	4	1	5	8	Liao et al. 2015
Caopuo	103.32	31.32	2120	67.9±8.9 *n* = 9	62.2±5.9 *n* = 35	4.0±1.7 *n* = 7	6.7±2.1 *n* = 25	5	2	11	9	Liao et al. 2015
Yele	102.20	28.92	2554	74.6±4.0 *n* = 43	62.5±3.6 *n* = 63	4.6±0.8 *n* = 43	3.1±1.1 *n* = 63	3	2	6	5	Liao et al. 2015
Baozigou	104.15	32.9	2452	90.0±5.7 *n* = 18	70.6±5.5 *n* = 28	8.3±1.3 *n* = 18	5.8±1.4 *n* = 28	6	3	11	8	Liao et al. 2015
Muyangcha ng	104.10	32.97	2640	91.9±7.9 *n* = 27	74.8±5.5 *n* = 47	8.4±1.8 *n* = 27	6.2±1.8 *n* = 47	4	3	11	11	Liao et al. 2015

Table S2. Environmental variables compiled to depict environment gradients for Andrew's toad (*B. andrewsi*).

Study sites	Annual Mean Temperature (℃)	Temperature Seasonality	Annual Precipitation (mm)	Precipitation of Driest Month (mm)	Precipitation Seasonality	UV-B Seasonality	Mean UV-B of Lowest Month
Gengda	11.471	686.730	899	7	83.769	105461	1269
Anning	16.892	497.813	946	5	100.332	137117	2134
Gucheng	13.600	469.453	796	11	62.191	223572	2801
Hanyuan	16.596	610.769	1214	13	95.051	139374	1541
Jinchuan	12.454	633.807	630	2	87.800	241349	2004
Jiulong	9.171	534.128	888	2	98.672	244765	2581
Luding	14.058	610.956	991	6	93.293	138801	1763
Lamasi	10.058	646.659	837	6	80.047	175943	1774
Maoxian	11.713	694.164	821	3	87.365	108096	1353
Puxiong	12.625	552.266	950	8	92.228	129969	1831
Shiziba	9.288	756.719	919	8	76.739	131824	1008
Taiping	14.600	472.546	954	8	96.214	137847	2204
Xunyangba	8.708	801.135	803	7	71.096	132526	1109
Yinchangou	9.046	646.700	787	5	79.754	139957	1580
Lingguan	14.563	685.569	1244	15	89.496	121027	1208
Muping	9.325	658.074	856	12	78.157	121027	1208
Yanjing	12.163	678.213	982	10	83.386	212128	1922
Dengcigou	10.525	665.564	869	10	78.696	212128	1922
Zhalangou	10.500	663.232	874	11	78.332	212128	1922
Church	9.167	660.968	836	11	77.954	212128	1922
Yaoji	8.850	632.100	744	6	81.661	231203	2092
Muli	0.754	545.533	843	4	92.031	243921	2597
Xingguqin	7.867	563.049	812	12	61.443	232966	2690

Qibie	11.313	549.376	856	11	62.806	232966	2690
Kegong1	11.125	545.575	905	12	62.541	232966	2690
Kegong2	11.454	542.048	937	12	63.014	232966	2690
Pantiange	11.750	533.189	931	13	61.709	228361	2718
Caopuo	8.742	662.368	795	6	78.569	114941	1475
Yele	9.363	540.619	930	6	92.354	227556	2435
Baozigou	5.688	716.139	729	4	76.994	152906	1449
Muyangchang	5.158	718.056	722	3	76.935	152906	1449

Figure S1. Pearson's correlation tests for bioclimatic variables and UV-B variables.

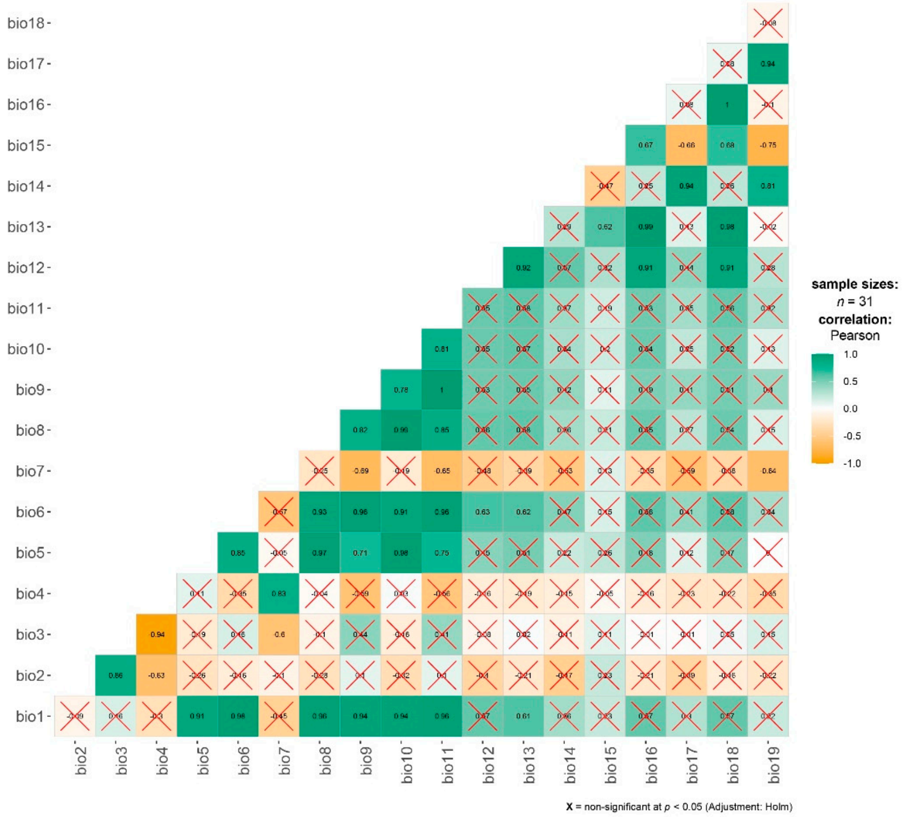

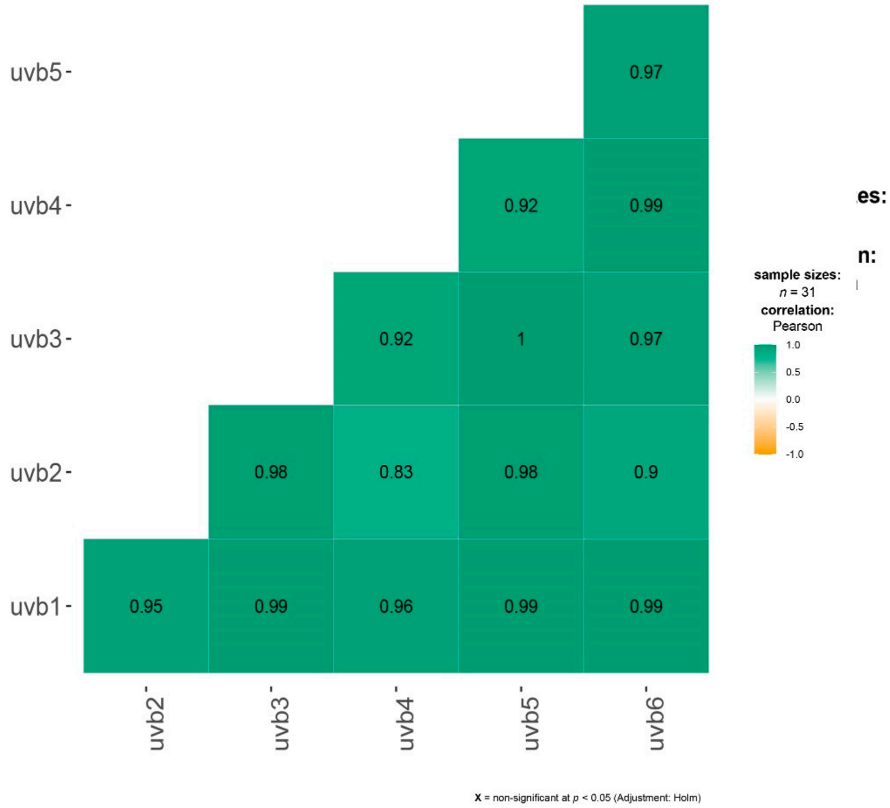

Supplement: Supplementary file 1 [file biology-11-01766-s001.zip › biology-2062333-supplementary.pdf]
